# Top 10 Public Health Challenges for 2024: Charting a New Direction for Global Health Security

**DOI:** 10.1002/puh2.70022

**Published:** 2024-12-30

**Authors:** Don Eliseo Lucero‐Prisno, Deborah Oluwaseun Shomuyiwa, M. B. N. Kouwenhoven, Thinley Dorji, Yusuff Adebayo Adebisi, Goodness Ogeyi Odey, Nsikakabasi Samuel George, Oluwatomisin Temidayo Ajayi, Olabode Ekerin, Emery Manirambona, Lin Xu, Joseph Christian Obnial, Adriana Miranda Viola, Isaac Olushola Ogunkola, Mohamed Mustaf Ahmed, Jerico B. Ogaya, Junjie Huang, Abraham Fessehaye Sium, Marcus Lester R. Suntay, William K. Chung, Pearl Irish V. De Paz, Hassan Sh Abdirahman Elmi, Omar Osman Hersi, Kebabonye P. Gabaake, Teresita Baricaua, Najib Isse Dirie, Prose Ivy G. Yepes, Donald John Wilson, Rossana Tofaeono‐Pifeleti, Ederson Delos Trino Tapia, Martin CS Wong

**Affiliations:** ^1^ Department of Global Health and Development London School of Hygiene and Tropical Medicine London UK; ^2^ Department of Health Promotion and Behavior College of Public Health University of Georgia Athens Georgia USA; ^3^ Global Health Focus Lagos Nigeria; ^4^ Department of Physics Xi'an Jiaotong‐Liverpool University Suzhou China; ^5^ Department of Internal Medicine Central Regional Referral Hospital Gelephu Bhutan; ^6^ College of Social Sciences University of Glasgow Glasgow UK; ^7^ Department of Health Policy London School of Economics and Political Science (LSE) London UK; ^8^ School of Medicine and Population Health University of Sheffield Sheffield UK; ^9^ Institute of Public Health Jagiellonian University Kraków Poland; ^10^ Department of Public Health State AIDS and STIs Control Program Ministry of Health Kaduna Nigeria; ^11^ School of Public Health University of Port Harcourt Port Harcourt Nigeria; ^12^ College of Medicine and Health Sciences University of Rwanda Kigali Rwanda; ^13^ Department of Thoracic Surgery The First Affiliated Hospital School of Medicine Zhejiang University Hangzhou Zhejiang China; ^14^ National Coalition of Independent Scholars Battleboro Vermont USA; ^15^ Global Health Focus Asia Bandung Indonesia; ^16^ YouthRise Dublin Ireland; ^17^ Faculty of Medicine and Health Sciences SIMAD University Mogadishu Somalia; ^18^ Department of Medical Technology Institute of Health Sciences and Nursing Far Eastern University Manila Philippines; ^19^ The Jockey Club School of Public Health and Primary Care Faculty of Medicine Chinese University of Hong Kong Hong Kong Hong Kong SAR China; ^20^ Department of Obstetrics and Gynecology St. Paul's Hospital Millennium Medical College Addis Ababa Ethiopia; ^21^ Office of the President World Surgical Foundation Manila Philippines; ^22^ Department of Biology Krieger School of Arts and Sciences Johns Hopkins University Baltimore Maryland USA; ^23^ Research and Development Office Biliran Province State University Naval, Biliran Philippines; ^24^ Department of Biology Amoud University Borama Somalia; ^25^ Faculty of Science Charles University Prague Czech Republic; ^26^ Office of the Vice Chancellor for Academics University of Burao Burao Somalia; ^27^ School of Allied Health Professions University of Botswana Gaborone Botswana; ^28^ Office of the President St. Paul University Iloilo Iloilo Philippines; ^29^ Department of Urology Faculty of Medicine and Health Sciences Dr. Sumait Hospital SIMAD University Mogadishu Somalia; ^30^ Office of the President Visayas State University Baybay, Leyte Philippines; ^31^ College of Medicine Nursing and Health Sciences Fiji National University Suva Fiji; ^32^ Faculty of Health Science National University of Samoa Apia Samoa; ^33^ Office of the Vice President for Academic Affairs University of Makati Makati Philippines

**Keywords:** artificial intelligence, climate fund, ethics, global health, health equity, pandemic accord, technology

## Abstract

As we navigate the complex landscape of global public health in 2024, this article provides a comprehensive exploration of challenges, ranging from infectious diseases, mental health, and substance use to environmental sustainability and emerging technologies. The aftermath of the COVID‐19 pandemic underscores the critical need to strengthen health systems, increase public financing, and foster effective international collaboration. The intricacies of global geopolitics, diplomacy, and public health highlight the importance of countries that do not address shared challenges through enhanced cooperative mechanisms and joint initiatives. From the imperative of global health security to the persistence of non‐communicable diseases and health disparities, this study delves into multifaceted issues, advocating for collective action, targeted interventions, and a commitment to prioritizing public health on a global scale. Focusing on addressing root causes and fostering equity, this study emphasizes the role of sustainable practices, community engagement, and intersectionality of research in building a resilient global health landscape. In this dynamic environment, 2024 calls for a unified global vision that encourages nations to collaborate more effectively to build a healthier and more resilient global community, ultimately paving the way for a future characterized by a shared commitment to public health challenges.

## Graphical abstract

The top 10 public health challenges for 2024 emphasize the need for a unified global approach to address infectious diseases, health disparities, climate change, mental health, substance use, reproductive health, food safety, emerging technologies, healthcare costs, and global collaboration.

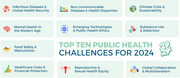

## 1 Introduction

Public health, an ever‐evolving field, plays a crucial role in safeguarding global communities’ well‐being. As we navigate in 2024, public health faces diverse challenges, including infectious and non‐communicable diseases (NCDs), environmental issues, and health disparities [1, 2]. To address these challenges effectively, understanding and prioritizing them is essential, forming the basis for developing policies, strategies, resource allocation, and research efforts to ensure global community health and safety. Public health challenges are complex and impact the entire population, with chronic diseases such as heart disease, cancer, and diabetes disproportionately affecting specific demographic groups, including racial and ethnic minorities, low‐income populations, and older adults. These conditions exacerbate health disparities and contribute to inequities in healthcare access and outcomes [3]. Emerging concerns such as substance use, mental health issues, health security, and the aftermath of the COVID‐19 pandemic [3] add to the complexity. Reproductive and sexual health challenges, malnutrition, and food safety issues further contribute to the multifaceted nature of public health. Poverty, coupled with social inequalities, exacerbates the burden of the disease.

In this era of constant shifts in health and medical sciences, understanding and anticipating challenges is essential for making informed decisions that significantly impact global health. This study highlights 10 key public health challenges for 2024: (1) infectious diseases and global health security, (2) NCDs and health disparities, (3) climate crisis and environmental sustainability, (4) mental health in the modern age, (5) substance use and addiction, (6) reproductive and sexual health equity, (7) food safety and malnutrition, (8) emerging technologies and public health ethics, (9) healthcare costs and financial protection, and (10) global collaboration and multilateralism. These challenges were selected on the basis of their global impact, urgency, and potential for significant public health intervention. In this study, the *Public Health Challenges* Editorial Team provides a comprehensive examination of these challenges (Table 1), shedding light on their implications and exploring potential solutions. By offering a forward‐looking perspective, this study aims to equip policymakers, researchers, and practitioners with insights for proactive and targeted responses. The ultimate goal is to strengthen the resilience of public health systems worldwide and promote the well‐being of communities on a global scale.

**TABLE 1:** Top 10 public health challenges for 2024 and their rationales.

Public health challenge	Rationale for inclusion
Infectious diseases and global health security	The COVID‐19 pandemic showed the devastating impact infectious diseases can have and highlighted the need for strong global health security measures, such as disease surveillance and robust healthcare systems
Non‐communicable diseases and health disparities	Non‐communicable diseases (NCDs) like heart disease and cancer continue to be a leading cause of death worldwide, particularly in low‐ and middle‐income countries. Health disparities exacerbate this issue by creating barriers to prevention and treatment for certain populations
Climate crisis and sustainability in environmental action	Climate change poses an imminent threat to human health and well‐being, with vulnerable populations and regions like small island nations facing disproportionate risks. Addressing the climate crisis requires a focus on environmental sustainability and climate justice to mitigate the health impacts
Mental health in the modern age	Mental health disorders, such as depression and anxiety, are increasingly prevalent globally. The COVID‐19 pandemic has further highlighted the importance of addressing mental health, as rates of loneliness and emotional distress have risen
Substance use and addiction	Substance use, including tobacco, alcohol, and drug use, continues to be a major public health concern globally, leading to a range of health and social problems. The vaping epidemic among youth and the ongoing efforts of the tobacco industry to undermine public health measures require continued attention
Sexual and reproductive health equity	Ensuring equitable access to sexual and reproductive health (SRH) information and services, including contraception and safe abortion, is crucial for individual and societal well‐being. Barriers to SRH, such as restrictive policies and social stigma, perpetuate health disparities
Food safety and malnutrition	Global food security is threatened by a confluence of factors, including conflict, climate change, and economic instability. These issues can lead to food shortages, price spikes, and malnutrition, particularly in vulnerable populations
Emerging technologies and public health ethics	The rapid advancement of technologies like artificial intelligence (AI) presents both opportunities and challenges for public health. Ethical considerations regarding data privacy, algorithmic bias, and equitable access to technology must be addressed
Healthcare costs and financial protection	The rising cost of healthcare and limited financial protection mechanisms pose a significant barrier to accessing essential health services, pushing many individuals and families into poverty. The COVID‐19 pandemic has exacerbated these challenges, highlighting the need for increased investment in health systems
Global collaboration and multilateralism	Addressing complex public health challenges requires effective global collaboration and multilateralism. Geopolitical tensions, conflicts, and the erosion of human rights pose significant obstacles to cooperation, underscoring the need for strong diplomatic efforts

## 2 Infectious Diseases and Global Health Security

The critical need for robust global health security has become increasingly apparent in the 21st century, particularly in the wake of the COVID‐19 pandemic [4]. The Independent Panel for Pandemic Preparedness and Response (IPPPR) in 2021 highlighted significant deficiencies in emergency response, disease surveillance, and health system resilience, underscoring the urgent need for a comprehensive reassessment of global preparedness and response mechanisms [4]. The IPPPR's recommendations call for stronger World Health Organization (WHO) authority, a new global surveillance system, and increased international financing for pandemic preparedness. Despite these global efforts, the world remains vulnerable to infectious diseases, underscoring the importance of enhanced collaboration to address emerging threats such as neglected tropical diseases (NTDs) and antimicrobial resistance (AMR) [5]. Continued evaluation of progress toward the 2030 targets of the End TB Strategy, zero deaths from rabies, and the elimination of human papillomavirus (HPV)‐associated cervical cancer remains critical. Global collaboration, as demonstrated by the lessons learned from COVID‐19, demands a unified commitment that can be reflected in a comprehensive pandemic accord or treaty [4]. Ensuring equitable global access to vaccines and bolstering health system resilience are essential components for mitigating the impact of future pandemics.

The COVID‐19 pandemic has revealed significant flaws in global health governance, particularly concerning equitable access to medical countermeasures. The IPPPR recommends transforming the current ACT‐A into a truly global, end‐to‐end platform for vaccines, diagnostics, therapeutics, and essential supplies [4]. This transformation requires revising regulations, establishing a pandemic treaty that prioritizes marginalized populations, and creating a WHO platform for medical countermeasures [4, 6]. Emphasis should be placed on boosting localized production and fostering regionally driven solutions for effective epidemic control. The treaty must comprehensively address future pandemic prevention, preparedness, and response, with a focus on prioritizing underserved populations and ensuring their meaningful participation in decision‐making processes [6]. Key considerations include the impact on minority communities, global health disparities, resource allocation for research, and the importance of community engagement in building a more resilient and equitable global health landscape.

Furthermore, the WHO's recognition of noma as an NTD marks a crucial step in addressing a severe and often fatal condition that affects malnourished children. This acknowledgment underscores a commitment to expanding health services for vulnerable populations, raising global awareness, catalyzing research, and stimulating funding for disease control [7]. Noma's inclusion in the NTD list highlights its deep connection to extreme poverty and malnutrition, emphasizing its broader social impact. This recognition should inspire global efforts to raise awareness, intensify research, and implement interventions for other NTDs, such as mycetoma, chromoblastomycosis, and other deep mycoses, as well as conditions like scabies and snakebite envenomation, particularly in low‐resource settings [7].

## 3 NCDs and Health Disparities

Despite global efforts to achieve Sustainable Development Goal (SDG) goal 3.4 on NCDs and mental health, NCDs remain a major global health issue. This burden is especially pronounced among the elderly and poses substantial financial costs to health systems worldwide. WHO estimates that NCDs cause 74% of global deaths, or 41 million people per year [8]. NCDs kill 5.5 million in the Americas and Caribbean each year, with 85% of fatalities in Low‐ and Middle‐Income Countries (LMICs) [9]. Studies predict that NCDs will outnumber communicable, nutritional, and maternal and perinatal fatalities in Sub‐Saharan Africa by 2030, with cardiovascular disease (CVD) alone rising from 1.3 million in 2004 to 2.5 million in 2030 [8]. Despite effective therapies and preventive measures, NCD‐related challenges persist. CVD is a major cause of death and disability in the United States [10].

Amid ongoing efforts to achieve universal health coverage, health disparities continue to exist across social groups globally, transcending development and income status. These disparities have profound social and economic ramifications for individuals and nations [11]. Refugees, migrants, indigenous people, individuals with disabilities, women, children, rural residents, and ethnic minorities face socioeconomic and structural barriers, discrimination, and inadequate healthcare access [8, 12]. The persistence of these challenges underscores the continued relevance of the Bamako Call to Action on Research for Health, which advocates for increased funding for health research in developmental aid. Despite the ratification of the Call by multiple countries, the recommendation to allocate at least 2% of national health budgets and at least 5% of external aid for health projects to research often remains unmet. This gap in research funding impedes efforts to effectively address NCDs and health disparities, particularly in LMICs, where the burden is greatest [13].

Eliminating structural barriers and establishing equitable healthcare access requires addressing these concerns within the framework of the social and commercial determinants of health [12]. Expanding early NCD diagnosis and reducing modifiable risk factors—such as tobacco use, alcohol consumption, physical inactivity, and poor diet—are essential strategies. These include implementing policies that regulate commercial influences on health behaviors, promoting health literacy and education, strengthening healthcare infrastructure, and fostering multi‐sectoral collaboration. Moreover, interventions should be tailored to specific populations and contexts, taking into account sociocultural norms and economic constraints. Prioritizing primary prevention strategies, such as promoting healthy lifestyles and early detection, not only improves health outcomes but also reduces the long‐term economic burden associated with NCD management. Revisiting and strengthening the commitments outlined in the Bamako Call to Action is a critical step forward. By investing more in research, we gain the insights needed to tailor interventions that truly work for communities facing the highest burdens. This renewed focus on research is not just about gathering data; it is about fueling strategies that confront NCDs head‐on and dismantle the inequities that have persisted for far too long.

## 4 Climate Crisis and Sustainability in Environmental Action

Climate change presents an unprecedented global threat, with the Intergovernmental Panel on Climate Change (IPCC) warning that global temperatures could rise by 1.5°C above pre‐industrial levels as early as 2030 [14]. This looming crisis demands urgent and coordinated international action to mitigate its far‐reaching effects on human health, ecosystems, and economies worldwide. The vulnerability of small island nations, such as those in the Caribbean and Fiji, highlights the urgency of climate justice. Home to approximately 65 million people, these nations face disproportionate risks [15]. By 2100, sea levels could rise by 0.5–2 m, threatening up to 90% of coastal areas in some island states. Additionally, Category 4 and 5 hurricanes in the Atlantic Basin have increased by 25%–30%/°C of global warming [16].

Africa, despite contributing only about 4% of global greenhouse gas emissions, bears a disproportionate share of climate change impacts [17]. The continent's high reliance on climate‐sensitive sectors, like agriculture, limited adaptive capacity, and geographical vulnerabilities, exacerbate its susceptibility [18]. The consequences include increased frequency and intensity of droughts, especially in the Sahel and Southern Africa, threatening food and water security; rising sea levels endangering coastal communities, with West African coasts experiencing some of the highest erosion rates globally [18]; and shifts in vector‐borne diseases, potentially expanding the range of malaria and other tropical illnesses [19]. Climate change further exacerbates challenges like poverty, conflict, and economic instability, with projections suggesting that by 2050, over 85 million people in Sub‐Saharan Africa could be displaced due to climate‐related stressors [20]. Recent seismic events, such as the 2024 earthquake and tsunami in Japan, underscore the ongoing and unpredictable nature of these threats, reinforcing the need for global efforts to confront them [21].

Addressing climate change and promoting environmental sustainability have become pivotal challenges demanding immediate attention. Over the past decade, action against climate change has shifted toward emphasizing climate justice, particularly the impact of global inaction on vulnerable communities [22]. COP28 in Dubai marked a significant milestone with the creation of the Loss and Damage Fund and the first global agreement to transition away from fossil fuels, aiming for net‐zero emissions by 2050 [23]. Key resolutions include commitments to accelerate renewable energy adoption and reduce emissions, though challenges remain in carbon markets and in advancing adaptation and finance initiatives [24]. Addressing methane emissions, integrating food systems into national climate commitments, and elevating the role of cities in climate action illustrate the urgency for countries to turn pledges into concrete actions. Securing the necessary financing to combat climate change effectively is crucial.

Transitioning to net‐zero emissions has emerged as a central theme in mitigating climate change's impacts, requiring sustained collaboration, accountability, and a commitment to cleaner energy alternatives. Environmental sustainability efforts must integrate governmental policies and civil society initiatives into comprehensive strategies. These strategies should include responsible resource management, biodiversity conservation, circular economies, sustainable consumption, and waste reduction. Governments must focus on policy development, regulation enforcement, and international cooperation, whereas civil society can drive grassroots activism, public education, and partnerships with the private sector to promote sustainable innovation. This synergy will create a holistic approach to addressing the multifaceted challenges of climate change and advancing environmental sustainability.

## 5 Mental Health in the Modern Age

In 2024, researchers, policymakers, and mental health practitioners will actively explore innovative techniques in global mental health research and practice in response to rising global concerns. Near the end of 2023, WHO established the Commission on Social Connections to address loneliness, a global public health issue [25]. WHO's focus on loneliness underscores its significant negative impact on mental health, as chronic loneliness is associated with an increased risk of depression, anxiety, and other mental health disorders. The WHO's commission's mandate targets loneliness and promotes social connection, demonstrating its commitment to reducing the negative consequences of loneliness and social isolation on physical health [25]. As emotional distress and depressive disorders have become public health issues, mental health is prioritized. Depressive disorders, the leading cause of “years lost to disability” in 2017, are now the WHO's leading cause of disability worldwide [25]. Lockdowns, physical isolation, and the shift to distant work and online schooling have increased loneliness rates worldwide during the COVID‐19 pandemic [26]. This increase in loneliness has exacerbated mental health challenges, highlighting the complex relationship between social isolation and psychological well‐being. Amid the growing recognition of global mental health, there is an increasing demand for comprehensive and inclusive mental health policies that consider all regions, age groups, and individuals, including key populations [27]. Studies have revealed that individuals with mental illnesses are disproportionately affected by the social determinants of health [28]. It is imperative that researchers actively explore evidence to inform the development and implementation of global mental health policies. These studies should emphasize the impact of unequal access to mental health services, with a heightened focus on the social and cultural factors influencing mental health development. Collaborative research is crucial in developing culturally sensitive and inclusive interventions [29].

Given the evolving landscape of public health and the dynamics of mental health, researchers must embrace life‐span approaches to mental health research. This perspective recognizes that mental health is influenced by biological, psychological, and social factors that interact dynamically over time [30]. By examining mental health through this lens, researchers can better understand the trajectories of different mental health concerns, identify critical periods for intervention, and develop more targeted and effective prevention and treatment strategies [31]. For example, this approach has been particularly valuable in understanding the long‐term impacts of early life experiences on adult mental health and in developing interventions that address mental health needs at different life stages [32]. A comprehensive and evidence‐based approach to mental health research and advocacy, integrating findings from neuroscience, genetics, and psychological science, is crucial to bridging the gap between basic sciences and the clinical application of these findings.

## 6 Substance Use and Addiction

The WHO's latest tobacco trends report provides a global snapshot of tobacco use, highlighting that approximately 1.25 billion adults currently use tobacco. Encouragingly, the data show a sustained decline in tobacco use rates, with the prevalence dropping to one in five adults in 2022, a significant improvement from one in three in 2000 [33]. This reflects notable progress in global tobacco control efforts [33]. However, new challenges have emerged with the rise in the use of vaping devices, non‐combustible tobacco products, and nicotine pouches. These products, popular among youth, have a gap in adequate regulation and comprehensive research on their long‐term health effects [34]. Although e‐cigarettes are seen as less harmful than traditional smoking, they still pose risks, including nicotine addiction and potential cognitive effects in adolescents [35]. This situation underscores the need for ongoing public health strategies and further research to fully understand these products’ impact and develop effective regulations.

Substance use and addiction intersect with health, education, and social issues, presenting severe risks to youth, including drug‐ and alcohol‐related violence, prescription drug overdoses, and accidents [36, 37, 38]. The chronic and relapsing nature of substance abuse, with relapse rates ranging from 56.8% to 81.8%, highlights its persistent global challenge [39]. Data from the UNODC World Drug Report 2023 show that around 296 million people used drugs in 2021, a 23% increase from 2011, emphasizing the growing global challenge of substance use and addiction [40]. The WHO report also underscores the importance of protecting children aged 13–15 from tobacco and nicotine products, pointing to the escalating costs associated with substance use, including criminal activities, healthcare, rehabilitation, decreased productivity, and judicial expenses. As we approach the 10th WHO Framework Convention on Tobacco Control (WHO FCTC) Conference of Parties in Panama, it is crucial to continue combating tobacco industry interference. Maintaining evidence‐based tobacco control measures must remain a global health priority with sustained international support.

Although tobacco use trends show progress, alcohol‐related issues remain a significant global health concern. Recent studies reveal an increase in alcohol consumption, particularly in emerging economies. In Thailand, an upper middle–income country, the alcohol market is controlled by an oligopoly, prompting efforts to integrate WHO's SAFER initiatives into the national agenda [41]. Alcohol is associated with approximately 200 diseases and at least 14 types of cancers [42, 43]. The 2016 WHO estimate attributed 29% of alcohol‐related deaths to oncological diseases, 20% to liver cirrhosis, and 19% to cardiovascular disorders [44]. Even moderate drinking does not seem to reduce mortality risk, challenging previous beliefs about alcohol's health benefits [44]. Alcohol misuse also significantly affects society; for instance, a study in New Zealand found that 17% of child maltreatment cases among Māori were linked to hazardous parental alcohol consumption [45]. Furthermore, alcohol use disorders often co‐occur with other mental health conditions, complicating diagnosis and treatment [46]. Effective public health strategies include stricter regulations on alcohol availability, increased taxation, and enhanced community‐based interventions for prevention and treatment [47]. However, challenges remain, particularly in regulating online alcohol sales and enforcing drinking and driving deterrence measures.

## 7 Sexual and Reproductive Health Equity

Addressing sexual and reproductive health and rights (SRHR) equity remains a critical challenge, as disparities in access to SRHR information and services persist across diverse populations, despite ongoing research and interventions [48]. In the dynamic field of public health, ongoing efforts to enhance access to reproductive health services, including comprehensive sex education, contraception, and abortion, are crucial for achieving universal health coverage [49]. A review of progress toward development goals at the 2023 UN General Assembly revealed a significant impact on health, well‐being, economic growth, poverty eradication, education, reduced inequalities, and environmental sustainability. However, universal access to sexual and reproductive health (SRH)‐care services faces hindrances due to the politicization of sex, gender, and reproduction [50].

Restrictions on reproductive freedom, including limited access to healthy abortions, infringe upon women and girls' bodily autonomy and their right to make informed decisions about sex and childbearing [51]. It is imperative for stakeholders in SRHR to prioritize studies and interventions addressing barriers for vulnerable groups, including geographical, socioeconomic, political, cultural, and technological factors. Gender‐based disparities significantly impact outcomes in SRHR, influenced by societal gender norms and unequal power dynamics, limiting individuals’ control over their SRH [48]. Addressing this requires an intersectional research approach to understand the unique challenges faced by communities of different genders. Although digital technologies offer a cost‐effective solution to enhance SRH services [52], concerns about a potential digital divide must be addressed. Prioritizing community engagement is crucial for promoting SRHR equitably [52].

Gender‐based violence (GBV) also remains a critical issue intrinsically linked to SRHR [53]. GBV, which includes physical, sexual, and psychological harm, significantly impacts an individual's ability to exercise sexual and reproductive rights [54]. For instance, intimate partner violence is associated with higher rates of unintended pregnancies, unsafe abortions, and sexually transmitted infections [55]. The COVID‐19 pandemic has exacerbated this issue, with many countries reporting increased rates of domestic violence during lockdowns [56]. Addressing GBV is crucial for achieving SRHR equity, requiring multi‐sectoral approaches that include legal reforms, community education, and integration of GBV screening and support services into SRH care.

## 8 Food Safety and Malnutrition

The global food system faces urgent challenges related to food safety and security, worsened by geopolitical conflicts, climate change, and regional instabilities. The 2020 High Level Panel of Experts (HLPE) highlights that, after a period of decline, hunger has risen since 2014 [57]. In 2019, nearly 690 million people were undernourished, and around 2 billion experienced moderate or severe food insecurity [57]. The COVID‐19 pandemic further intensified food insecurity, potentially adding 83–132 million people to the undernourished population in 2020 [57]. The ongoing wars have disrupted food supply chains, especially for grains and oilseeds, affecting global food security and exacerbating malnutrition among vulnerable populations [58]. Conflicts in regions like Sudan, the Democratic Republic of the Congo (DRC), and the ongoing strife in Israel and Gaza have similarly disrupted local food production and distribution, worsening malnutrition [59, 60].

Climate change compounds these issues, as seen in the Sahel, where altered weather patterns and environmental degradation threaten agricultural productivity and increase vulnerability to malnutrition [61]. Food safety is also crucial, as unsafe foods undermine proper nutrition and health. The HLPE report emphasizes that food safety is integral to food systems, impacting health, nutrition, food security, and economic development [57]. Foodborne diseases affect millions globally each year, with the greatest burden on vulnerable populations in low‐ and middle‐income countries [57].

Addressing these challenges requires a strong commitment to food safety and combating malnutrition through global health policies. This includes strengthening food systems, promoting sustainable agriculture, and ensuring equitable access to food resources. Climate‐resilient agricultural practices, such as sustainable farming, water conservation, and drought‐resistant crops, are vital for enhancing food system resilience. The HLPE report recommends integrating food safety and security concerns by improving food safety governance and regulation, promoting sustainable agricultural practices, enhancing post‐harvest handling and storage, increasing food safety education, and investing in research and innovation [57]. Sustainable projects that focus on resource management and biodiversity are essential for addressing environmental degradation, which both results from and contributes to conflict. Policies that prioritize environmental goals, such as preserving high‐quality habitats, can help resolve conflicts between agriculture and the environment. Substantial investments in conflict prevention, peacebuilding, and sustainable development are crucial for creating a supportive environment for global food security and improved nutrition.

## 9 Emerging Technologies and Public Health Ethics

In the rapidly evolving landscape of technological advancements, particularly in artificial intelligence (AI), unique insights underscore the delicate balance between innovation and ethical principles as we traverse the pivotal year 2024. The integration of AI, biotechnology, and groundbreaking technologies is not merely a technological leap but also a critical driver in shaping a sustainable and equitable future [62]. Examining the medical field reveals the promise of large language models (LLMs), such as ChatGPT, offering a glimpse into the potential of predicting medical outcomes and generating responses. The ongoing debate surrounding the clinical reliability of models such as GPT‐3.5, with its strengths aligning with cancer treatment guidelines but exhibiting occasional “hallucinations,” adds a nuanced layer to the narrative [63].

Despite these challenges, transformative power lies in democratizing medical expertise globally, especially for underserved populations, and envisioning AI as a vehicle for delivering optimal healthcare on a global scale. This vision resonates with concerns raised about safeguarding health information in the surge of digital health technologies, where the ethical imperative of ensuring data security and privacy is a profound necessity [62, 64]. The confluence of emerging technologies and public health ethics necessitates proactive measures to navigate the evolving work landscape and strike a balance between technological advancements and human‐centric values. Ethical considerations become the guiding force in policies, shaping a future in which technology augments human potential without displacing it [62, 64]. Within the realm of public health, the magnification of ethical implications underscores the importance of transparency and accountability, fostering a future in which innovation seamlessly aligns with the values of equity, accountability, and responsible progress. This integrated narrative envisions a future of innovation that not only harnesses the transformative power of AI but also harmonizes it with the values of a healthier, fairer, and ethically grounded global community.

## 10 Healthcare Costs and Financial Protection

The lessons learned from the COVID‐19 pandemic have highlighted the importance of investing in strong health systems. Despite the initial progress in global health coverage and increased financial commitments to health, sustaining these gains has been challenging post‐pandemic [65]. Financial strain on nations has led to reduced government health spending and higher out‐of‐pocket payments for healthcare worldwide [66]. A report from the WHO, International Bank for Reconstruction and Development (IBRD), and World Bank (2023) reveals this aftermath, indicating that approximately 2 billion people face financial hardship due to catastrophic and impoverishing health spending, a stark increase beyond pre‐pandemic rates, reaching approximately 13.5% globally in 2019 [67]. Regional disparities persist, with significant increases in impoverishing health spending observed in Sub‐Saharan Africa, Asia, Latin America, the Caribbean, and Europe. Notably, the European region experiences varying degrees of impoverishing health spending, ranging from 1% to over 7% of households, and catastrophic health spending ranging from uder 2% to over 14% of households across countries [68]. This financial burden disproportionately affects the poorest households in both developed and developing nations. The surge in healthcare costs also reflects growing unmet health needs, as individuals struggle to access essential healthcare after the pandemic. The increasing aging population raises important questions about future global health financing requirements that demand proactive measures, such as increased public funding for health along with effective execution rates and accountability mechanisms for managing limited resources wisely. Low‐ and middle‐income countries must prioritize sustainable domestic financing through mechanisms such as health taxes, while simultaneously reducing co‐payment rates, particularly where voluntary insurance systems exist, thereby making affordable healthcare more accessible overall.

## 11 Global Collaboration and Multilateralism

Global geopolitics, diplomacy, and public health will pose significant challenges in 2024 due to conflicts, populism, human rights erosion, climate change, migration, and security concerns [69]. Over the past two decades, global health diplomacy and foreign policies have evolved in response to the changing power dynamics among nations. As we navigate a world shaped by geopolitical tensions, international collaboration becomes crucial in addressing the complex challenges that cross borders. The shifting distribution of power has greatly influenced global health policies and initiatives. Ongoing crises such as the Middle East conflict and Russia–Ukraine tensions underscore the intricate relationship between geopolitical instability and public health challenges [70]. Furthermore, negotiations for a pandemic highlight the importance of multilateral collaboration to safeguard global health security. The year 2024 is designated as a global election year that adds another layer of complexity by emphasizing the need to analyze how electoral processes may impact health policies [71].

Certain strategies are crucial to enhance global health collaboration. Recognizing mutual interdependence and sharing vital information form the basis of collaboration. Integrating health considerations into foreign policies and closely monitoring electoral processes can ensure that health remains a priority at the global stage [69]. Investing in public health infrastructure and strengthening multilateral institutions, such as the WHO and the UN, enables nations to collectively address health challenges. Promoting evidence‐based decision‐making, empowering non‐governmental organizations, and fostering cross‐sector partnerships are essential for promoting health equity and resilience amid changing geopolitical landscapes. Effective collaboration requires active local involvement, mutual respect, and sustained dedication in order to advance shared interests.

## 12 Conclusion

In 2024, the global public health landscape faces complex challenges requiring resilient health systems, increased public funding, and stronger international collaboration. The aftermath of COVID‐19 has highlighted the need for sustained investments in public health to address financial strain and reduce out‐of‐pocket payments. Key areas, like global health security, mental health, substance use, food safety, environmental sustainability, and emerging technologies, demand coordinated action. Governments must collaborate in resource sharing, capacity building, and equitable health policy development to reduce disparities and strengthen global health resilience. Achieving this requires innovation, evidence‐based policies, and sustainable practices across sectors. A unified global approach rooted in cooperation and mutual support is essential for confronting public health challenges. This shared commitment will help build a more resilient, equitable, and healthier global society, ensuring public health remains a top priority worldwide.

## Author Contributions

D.E.L.P. conceptualized the study. D.O.S., N.S.G., O.E., and O.T.A. prepared the first draft of this manuscript. All authors have reviewed and approved the final manuscript. All authors contributed to manuscript review and approved the final version of the manuscript.

## Ethics Statement

This study did not involve any human or animal subjects and, thus, did not require review by an Institutional Review Board (IRB).

## Conflicts of Interest

Don Eliseo Lucero‐Prisno III and M. B. N. Kouwenhoven are members of the Editorial Board for Public Health Challenges and have contributed as co‐authors to this article. Deborah Oluwaseun Shomuyiwa, Thinley Dorji, Goodness Ogeyi Odey, Adriana Viola Miranda, Isaac Olushola Ogunkola, Yusuff Adebayo Adebisi, Junjie Huang, Lin Xu, and Joseph Christian Obnial are members of the Youth Editorial Board for Public Health Challenges and have also contributed as co‐authors. To minimize potential bias, these individuals were excluded from all editorial decision‐making processes related to the acceptance of this article for publication.

## Data Availability

Data sharing is not applicable to this article, as no new data were created or analyzed in this study.
